# Changes in Emotional and Vocal Expression in Job Interview Simulations with an AI-Enhanced Chatbot for University Students

**DOI:** 10.3390/bs16091554

**Published:** 2026-09-02

**Authors:** Alberto Grajeda, Pamela Cordova, Juan Pablo Cordova, María Isabel Pueyo, Patricia Gasser, Isabel La Fuente, Hernán Naranjo

**Affiliations:** 1 Research Center for Innovation in Information Technology for Education, Universidad Privada Boliviana, Cochabamba, Bolivia; agrajeda@upb.edu; 2 Center for Research in Economics and Entrepreneurial Sciences, Universidad Privada Boliviana, Cochabamba, Bolivia; 3 Applied Neuroscience Laboratory, Universidad Privada Boliviana, Cochabamba, Bolivia; jpcordova@upb.edu; 4 Olave School of Business and Faculty of Business Sciences and Law, Universidad Privada Boliviana, Cochabamba, Bolivia; mpueyo@upb.edu (M.I.P.); patriciagasser@upb.edu (P.G.); isabellafuente@upb.edu (I.L.F.); hnaranjo@upb.edu (H.N.)

**Keywords:** AI-powered chatbot, facial-expression patterns, vocal-expression patterns, simulated job interviews, university students, applied neuroscience

## Abstract

This study examined whether AI-powered chatbot-based training was associated with changes in university students’ Facial and Vocal Emotional Reaction Time Proportions during simulated job interviews. A one-group pretest–posttest design was conducted with 54 third- and fourth-year students enrolled in a Human Talent Management course at a private Latin American university. This study was implemented in an Applied Neuroscience Laboratory using iMotions-supported facial-expression recognition and vocal analysis technologies. Participants first completed a baseline simulated interview, followed by three chatbot-based training sessions using HR-expert-validated questions, end-of-session scoring, and qualitative feedback. A final simulated interview was then conducted to compare pre- and post-training indicators. Facial emotional reaction time was analyzed through aggregate indicators—positive, negative, neutral, confusion, and sentimentality—and specific facial-expression categories, including joy, surprise, anger, sadness, disgust, fear, and contempt. Vocal emotional reaction time was examined through happiness, sadness, anger, and neutrality. Pre–post differences were assessed using paired-samples *t*-tests and complementary Wilcoxon signed-rank tests. Positive facial emotional reaction time increased significantly from 3.52% to 14.75%, with a mean increase of 11.23 percentage points, 95% CI [4.79, 17.67]. Facial joy increased significantly from 2.38% to 10.10%, with a mean increase of 7.72 percentage points, 95% CI [3.30, 12.14], while vocal happiness increased significantly from 2.79% to 10.71%, with a mean increase of 7.92 percentage points, 95% CI [3.38, 12.46]. Each of these principal outcomes showed a standardized paired effect of *d_z_* = 0.48, 95% CI [0.19, 0.76], with corresponding Wilcoxon effect-size estimates ranging from *r* = 0.40 to *r* = 0.44. Several negative and neutral indicators also decreased after training; however, their mean-based standardized effects were generally smaller and some statistically significant findings were supported primarily by the Wilcoxon signed-rank test. Overall, chatbot-based interview training was associated with changes in algorithmically classified facial and vocal-expression patterns and may provide a complementary tool for structured interview practice in higher education.

## 1. Introduction

Artificial intelligence has reshaped talent acquisition over the past decade. Resume screening, candidate profile analysis, real-time interview interpretation, and interview-preparation training are increasingly mediated by AI-driven systems, altering hiring dynamics for recruiters and applicants alike (Callejas et al., 2014; Nuzula & Amri, 2023). Beyond operational gains, AI enables the standardization of evaluation criteria, reduces inconsistencies among interviewers, and delivers evidence-based feedback, benefits that extend to educational institutions by allowing students to be trained in professional life skills (Callejas et al., 2014).

These tools are not without risks when applied to actual hiring decisions. The European Artificial Intelligence Regulation (EU 2024/1689) prohibits the use of AI systems to infer emotions in the workplace, deeming it intrusive to privacy and human dignity in structurally asymmetric relationships (European Parliament & Council of the European Union, 2024). Accordingly, the present study does not propose these tools as a candidate selection mechanism, but as a pedagogical training instrument that allows students to receive feedback and strengthen their skills for real job interviews.

Among the most practical applications of this shift is the use of AI chatbots as interview-simulation environments, which allow students to rehearse responses repeatedly, free from the social pressure or evaluative anxiety that often distorts performance in real settings (Roulin et al., 2019). Verbal clarity, argumentative coherence, and non-verbal communication management, long identified as decisive in interview outcomes (Lievens & de Paepe, 2004), can thus be refined through structured, low-stakes practice. However, technology-mediated interviews tend to elicit lower psychological pressure than face-to-face interviews (Kleinlogel et al., 2023), and automatically generated feedback may contain errors or reproduce discursive biases derived from training data, requiring critical review by the user (Brubacher et al., 2025).

This kind of preparation is especially relevant for university students and early-career professionals, who frequently enter selection processes with limited exposure to high-pressure evaluation contexts. Recruiters consistently rank emotional intelligence, stress management, and collaborative ability among the most valued competencies in candidates (Howe, 2014), yet these are precisely the skills most vulnerable to anxiety. Negative emotional states, including fear and apprehension, are well-documented responses to evaluative pressure and represent concrete barriers to authentic self-presentation (Brunet & Müller, 2024; Shen, 2023; Xu, 2023).

Despite the growing adoption of AI chatbots for interview preparation, empirical evidence on whether such training is associated with observable changes in students’ facial and vocal expression during simulated interviews remains scarce, particularly in Latin American higher education. To address this gap, this study used facial-expression recognition and vocal analysis technologies to quantify university students’ facial- and vocal-expression patterns before and after AI-powered chatbot-based training, examining the magnitude and precision of the pre–post changes through paired comparisons, 95% confidence intervals, and effect-size estimates.

## 2. Literature Review

### 2.1. Recruitment Interviews in a Digital and AI-Mediated Context

Globalization, digitalization, and the disruptions brought about by the pandemic have compelled human resources departments to rethink their talent attraction and selection models, incorporating technological tools capable of managing growing volumes of applicants with greater efficiency and reduced bias (Mendoza Armijos, 2021). Organizations have progressively adopted automated systems for resume screening, interview scheduling, and preliminary competency assessment, driven by the demand for agility and precision in selection processes (Piedra Mayorga et al., 2023). This convergence has raised the bar for candidates, who are now expected to demonstrate their capabilities in environments mediated by screens, algorithms, and automated evaluation systems (Mendoza Armijos, 2021; Piedra Mayorga et al., 2023).

The job interview remains one of the most consequential moments in any selection process, as it allows recruiters to observe how a candidate communicates, handles pressure, and adapts to an unfamiliar interactional context (Mocha Román, 2018). Emotional intelligence, understood as the capacity to perceive, understand, and regulate one’s own emotions and those of others, has emerged as a meaningful predictor of success in high-stakes evaluative contexts (Howe, 2014). The emotional states a candidate experiences during an interview shape performance directly, influencing the quality of responses, body language, and the overall impression conveyed to the evaluator (Shen, 2023; van Doorn et al., 2015). Personality traits such as perfectionism or fear of failure can intensify anxiety responses even in technically competent candidates (Hogan et al., 2011), which is why the development of soft skills such as stress management, active listening, empathy, and assertive communication has become a priority for candidates and for the institutions that prepare them (Roulin et al., 2019).

The consolidation of remote and hybrid work has accelerated the migration from in-person to virtual interviews (Callejas et al., 2014). Asynchronous technology-mediated interviews, in which candidates respond to pre-recorded questions without a live interviewer, substantially alter the emotional and communicative dynamics of the process (Hemamou et al., 2019; Sardi & Troilo, 2020) and elicit emotional responses that differ markedly from those observed in face-to-face settings (Tilston et al., 2024). Artificial intelligence has also begun to function as an active evaluative agent, capable of analyzing responses, detecting behavioral patterns, and generating candidate profiles (Albassam, 2023; Upadhyay & Khandelwal, 2018). At the same time, research has identified limits of AI-based training: chatbots generate controlled practice environments but do not necessarily reproduce the peaks of psychological pressure of a real selection process before human evaluators (Daryanto et al., 2024); language models operate probabilistically and may reproduce latent discursive biases or generate factual inaccuracies (Lund et al., 2023); and automated emotional analysis should be regarded as an indicative and complementary measure, since algorithmic coding processes affective expression through standardized categories that do not fully capture the contextual complexity of human communication (Brubacher et al., 2025). These tools are therefore most effective when designed under rigorous pedagogical approaches and conceived as complementary resources within the preparation process.

### 2.2. Multimodal Analysis Technologies in Interview Evaluation

The emergence of multimodal analysis tools has equipped recruitment systems to capture and process information beyond the verbal content of candidates’ responses. Natural Language Processing (NLP) software makes it possible to evaluate, in real time, the tone, argumentative clarity, and relevance of what candidates say, identifying linguistic patterns associated with competencies such as confidence, empathy, and problem-solving ability (Huang & Rust, 2018). This analytical capacity transforms the interview into a measurable, scalable event, substantially reducing the subjectivity that has historically characterized human evaluation.

Complementing these linguistic tools, facial-expression analysis and voice analysis can provide additional information about observable non-verbal behavior during interviews. Facial-expression systems detect visible facial configurations and classify them into predefined expression categories, while eye-tracking technology can characterize patterns of visual attention in response to interview stimuli (Balconi & Cassioli, 2022). Voice analysis can quantify acoustic and prosodic characteristics such as pitch, tone, volume, speaking rate, and intonation. These outputs may be useful for describing observable facial- and vocal-expression patterns, but they should not be interpreted as direct measures of confidence, emotional stability, or other internal psychological states. Their meaning remains dependent on the algorithm, communicative context, and measurement conditions.

### 2.3. Theoretical Framework: AI Chatbots, Experiential Learning, and Feedback

Chatbot-based interview preparation represents a meaningful departure from traditional readiness methods. Unlike static resources such as written guides or instructional videos, chatbots are capable of sustaining dynamic, responsive conversations, adapting in real time to the user’s answers and delivering personalized feedback at each interaction (Dixit et al., 2022). This interactional capacity enables the development of the interviewee by simulating the evaluative reasoning of an experienced recruiter.

The analogy with a talent management specialist is well-founded. These systems are calibrated against criteria established by human resources professionals, enabling them to pose structured questions, assess the relevance of responses, and generate feedback cycles oriented toward continuous improvement (Chamorro-Premuzic, 2017). According to Zhou et al. (2019), research on chatbots used to evaluate candidates can infer personality traits from response patterns, offering a complementary perspective on candidate profiles that goes beyond what self-reported assessments typically capture; however, the EU has regulated the use of these resources as predictors of personality and behavior. Furthermore, their round-the-clock availability and their fundamentally non-evaluative nature—in the sense that interactions carry no real-world consequences for the candidate—make them accessible training tools, free from the social pressure that tends to distort performance in actual interviews (Nawaz & Gomes, 2019).

The practical value of chatbot-based simulations lies in the opportunity for deliberate, repeated practice that they afford. The ability to complete multiple rounds of simulated interviews, receive specific feedback after each attempt, and progressively refine one’s responses supports the development of both communicative fluency and emotional regulation within a controlled environment (Roulin et al., 2019; Stephens et al., 2021). This iterative process is particularly valuable for university students and early-career professionals, who frequently enter selection processes with limited prior exposure to high-pressure evaluative settings. Chatbot-based simulations can replicate different interview styles—structured, situational, or competency-based—and adjust their difficulty level to match the candidate’s developmental stage, making them versatile instruments for personalized preparation (Dixit et al., 2022). When voice and emotional analysis are incorporated, these simulations extend their feedback to non-verbal dimensions of performance—voice modulation, eye contact, and facial expression—aspects that candidates rarely have the opportunity to observe and address on their own (Batrinca et al., 2013). This multimodal feedback loop meaningfully amplifies the formative impact of practice, integrating the content of responses with the manner in which they are delivered into a single, coherent learning cycle.

This training approach finds a solid theoretical anchor in Kolb’s experiential learning model (Kolb, 2015), which conceives learning as a cyclical process of four interconnected stages: concrete experience, reflective observation, abstract conceptualization, and active experimentation. In the context of chatbot-based interview preparation, the first simulated interview represents the concrete experience—the participant’s initial encounter with an evaluative situation—while the subsequent feedback from the chatbot constitutes the reflective observation phase, offering a structured opportunity to identify strengths and areas for improvement. The successive practice rounds correspond to abstract conceptualization and active experimentation, during which participants incorporate the recommendations to refine their vocal modulation, emotional expression, and response quality.

This iterative process aligns with the conception of Lipnevich and Panadero (2021), for whom feedback-based learning—which the chatbot enables—is not a unidirectional act of information transmission, but rather a cyclical process in which the student cognitively and affectively processes the information received, generating internal responses that produce internal feedback. The effectiveness of this process depends on the feedback delivered by the chatbot enabling the participant to address the key questions proposed by Hattie and Timperley (2007): “Where am I going?”, “How am I going?”, and “Where to next?” By consistently and specifically informing participants about which aspects of their responses can be improved, the chatbot guides them in progressively reducing the gap between their initial answers and the expected standard. In this way, each practice iteration brings the trainee closer to better outcomes in a future job interview, transforming feedback into an engine of continuous improvement. Thus, the post-training interview closes the experiential cycle by reflecting the consolidation of learning, while the interaction between the feedback received, the participant’s disposition to interpret it, and their capacity to act upon it determines the success of the training. This alignment between the proposals of Kolb (2015), Lipnevich and Panadero (2021), and Hattie and Timperley (2007) reinforces the validity of the experiential approach and explains the mechanisms through which iterative practice with a chatbot produces observable improvements in the trainee’s performance.

### 2.4. Empirical Evidence and Hypothesis Development

The evidence on chatbot-based interview preparation points consistently toward measurable improvements across multiple performance dimensions. Prior research has documented gains in vocal modulation, response clarity, emotional stability, and the ability to handle unexpected questions—competencies that recruiters routinely weight heavily in their evaluations (Roulin et al., 2019; Stephens et al., 2021). These findings suggest that structured AI-assisted training does more than prepare participants technically; it reshapes their emotional orientation toward the selection process itself.

Among the most significant outcomes of chatbot-based training is the development of self-confidence—an effect whose implications extend well beyond the immediate interview. A participant who has rehearsed their responses across multiple simulated scenarios, received detailed feedback on both their verbal and non-verbal communication, and had the opportunity to correct mistakes in a consequence-free environment arrives at a real interview with a psychological foundation that substantially changes how they experience the encounter (Chamorro-Premuzic, 2017; Zhang et al., 2022). This shift is not incidental. Perceived self-efficacy directly influences the quality of communication, the capacity to manage stress in the moment, and the overall impression a candidate makes on the evaluator. In this light, AI chatbots designed for interview preparation function not only as training tools but as instruments of personal development—ones that can help level the playing field for participants with varying levels of prior experience and exposure to formal selection processes (Albassam, 2023; Nawaz & Gomes, 2019).

One of the most consistently documented benefits of this type of training is its capacity to reduce the anxiety and tension that people typically associate with evaluation. By offering an environment entirely free of real-world consequences, chatbots dissolve much of the social pressure inherent in traditional interviews, allowing participants to focus on skill development without the emotional weight of external judgment (Howe, 2014). This reduction in affective load creates a more cognitively receptive state—one in which feedback can be absorbed and integrated far more effectively than it would be under stress.

The cognitive feedback that chatbots provide operates as the closing mechanism of the learning cycle, connecting observed performance to concrete, actionable recommendations. This process transforms emotion—often disruptive in evaluative contexts—into useful information for improvement, strengthening the candidate’s self-awareness and capacity for self-regulation (Shen, 2023). As participants accumulate practice experiences accompanied by constructive feedback, their perceived self-efficacy grows, setting in motion a productive cycle in which confidence reinforces performance and performance reinforces confidence. This dynamic is especially relevant for candidates prone to perfectionism or with a history of evaluative anxiety, who may find in chatbot-based training a structured space to recalibrate their relationship with assessment before facing the pressures of a real selection process (Hogan et al., 2011; van Doorn et al., 2015).

Taken together, experiential learning theory (Kolb, 2015), feedback theory (Hattie & Timperley, 2007; Lipnevich & Panadero, 2021), and the empirical evidence reviewed above suggest that repeated chatbot-based practice with feedback should be accompanied by more positive and less negative expressive patterns during interviews. Specifically, the reported gains in confidence and emotional stability (Chamorro-Premuzic, 2017; Roulin et al., 2019; Stephens et al., 2021; Zhang et al., 2022) support the expectation of higher positively classified facial and vocal expression after training (H1 and H2); the documented reduction of evaluative anxiety and negative emotional states (Howe, 2014; Shen, 2023; Xu, 2023) supports the expectation of lower negatively classified indicators (H3); and the greater expressive variation associated with engaged, feedback-informed practice (Batrinca et al., 2013; Dixit et al., 2022) supports the expectation of lower neutral classifications (H4).

### 2.5. Research Question and Hypotheses

The present study examines pre–post changes associated with AI-powered chatbot-based training in university students’ facial- and vocal-expression patterns during simulated job interviews designed to approximate the evaluative conditions of selection processes. Outcomes were operationalized as the Facial Emotional Reaction Time Proportion (%) and the Vocal Emotional Reaction Time Proportion (%), as defined in Section 3. Within this framework, this study poses the following research question:

To what extent is AI-powered chatbot-based training associated with pre–post changes in university students’ facial and Vocal Emotional Reaction Time Proportions during simulated job interviews, and what is the magnitude of these changes?

To address this question, the following hypotheses are proposed:

**H1.**  *Positive facial emotional reaction time, particularly facial joy, will be significantly higher after AI-powered chatbot-based training than at baseline.*

**H2.**  *Vocal happiness will be significantly higher after AI-powered chatbot-based training than at baseline.*

**H3.**  *Negative facial emotional reaction time and selected negatively classified facial and vocal indicators, particularly contempt, disgust, facial sadness, vocal sadness, and vocal anger, will be significantly lower after AI-powered chatbot-based training than at baseline.*

**H4.**  *Neutral facial emotional reaction time and vocal neutrality will be significantly lower after AI-powered chatbot-based training than at baseline.*

## 3. Methods and Materials

### 3.1. Research Design

This study followed a one-group pretest–posttest design. The same group of students was assessed before and after an AI-powered chatbot-based training intervention. No control group was included. Therefore, this study was designed to examine within-participant changes associated with the intervention, rather than to establish definitive causal effects.

The within-participant comparison factor was measurement occasion (pre-training versus post-training). The dependent variables were the Facial Emotional Reaction Time Proportion (%) and the Vocal Emotional Reaction Time Proportion (%), analyzed through the aggregate and specific indicators described in Section 3.8.

This design was appropriate for the purpose of this study because the primary objective was to examine within-participant pre–post changes in Facial and Vocal Emotional Reaction Time Proportions following a structured chatbot-based training process. Accordingly, the findings are interpreted as pre–post changes associated with chatbot-based training, rather than as causal effects attributable exclusively to the intervention.

### 3.2. Participants

Participants were 54 third- and fourth-year students enrolled in a Human Talent Management course at a private Latin American university. The course was selected because its content is directly related to recruitment, selection, professional communication, and job interview preparation, which made the simulated interview activity academically relevant. All participants completed the full protocol, including the baseline simulated interview, the three chatbot-based training sessions, and the post-training simulated interview. No a priori power analysis was conducted; the analytical sample comprised all eligible students who completed the protocol, and this study should therefore be interpreted as exploratory.

### 3.3. Ethical Considerations and Data Protection

The research protocol was reviewed by the Ethics Committee in Research at Universidad Privada Boliviana and classified as exempt from formal ethical evaluation because it involved a minimal-risk, non-invasive educational training activity based on simulated job interviews. Participation was voluntary; students were informed about the academic purpose of this study and the use of facial, vocal, and chatbot-interaction data for research purposes, and informed consent was obtained from all participants. The database was de-identified before analysis, and results were reported only in aggregated form.

### 3.4. AI-Powered Chatbot Design and Calibration

The AI-powered chatbot was designed to reproduce a structured interview environment and provide students with end-of-session feedback on the quality of their answers. Training was organized around the following five fixed questions, presented to each participant one at a time and in the same order:

- What has been your greatest professional failure, and what did you learn from it?
- What are your salary expectations, and how do you justify them?
- What are your main strengths and weaknesses?
- Tell me about a difficult problem you faced and how you solved it.
- Why should we hire you instead of another candidate?

These questions addressed five common dimensions of employment interviews: reflective learning from failure, salary expectations and professional self-positioning, self-awareness regarding strengths and weaknesses, problem-solving ability, and articulation of the candidate’s professional-value proposition. Ten human resources experts reviewed the questions for professional relevance, clarity of wording, realism of the simulated situation, and usefulness for evaluating students’ communicative performance (Table 1).

The training was conducted using ChatGPT-4o (OpenAI), through its customizable GPT functionality. A tailored GPT was configured to simulate a job interviewer by presenting the validated questions, evaluating participants’ responses, and providing qualitative feedback in each session. Participants accessed the chatbot through a link provided by the research team and interacted exclusively through the voice interface, in Spanish, to reproduce the oral and real-time dynamics of a job interview; session recordings were reviewed to verify that training was completed through synchronous vocal interaction.

To support replicability, the principal configuration instructions of the customized GPT are reported below. The prompt starts as follows: “Act as a strict and demanding employer conducting a high-pressure job interview. Maintain a challenging and mildly confrontational interview style while remaining professional and respectful toward the candidate”.

#### 3.4.1. Interview Sequence

Begin the interview immediately, without providing introductory explanations. Ask the following five questions one at a time and wait for the candidate’s response before presenting the next question:

What has been your greatest professional failure, and what did you learn from it?

What are your salary expectations, and how do you justify them?

What are your main strengths and weaknesses?

Tell me about a difficult problem you faced and how you solved it.

Why should we hire you instead of another candidate?

Do not evaluate, correct, praise, or comment on the candidate’s answer immediately after each question. Complete the full sequence of five questions before providing any evaluation.

#### 3.4.2. End-of-Session Feedback

After the candidate has answered all five questions:

- Assign each answer a score from 0 to 10.
- Briefly explain the main strengths and weaknesses of each answer.
- Provide concrete recommendations for improving the content, clarity, organization, relevance, and professional presentation of the responses.
- Be critical and demanding when assigning the scores.
- Calculate the overall session score as the arithmetic mean of the five individual scores, expressed on a 0-to-10 scale.

When the candidate responds using voice input, provide qualitative comments on audible aspects of delivery, such as speech clarity, pace, volume, intonation, hesitations, and vocal variation. Do not make clinical or diagnostic claims about the candidate’s emotional or psychological state.

#### 3.4.3. Repeated Practice Cycles

After completing the feedback, begin another interview cycle using the same five-question structure. At the end of each subsequent cycle, provide the same individual scores, qualitative recommendations, and overall average.

From the second cycle onward, compare the current performance with the previous cycle. Identify the aspects in which the candidate improved, remained stable, or performed less effectively. The comparison should consider both the quality of the answers and, when voice responses are available, observable characteristics of vocal delivery.

The chatbot thus followed a delayed-feedback structure: scores (0–10 per response) and qualitative feedback were generated only after the full five-question cycle, preserving the continuity and evaluative pressure of the interview. The assessment was holistic, considering clarity, coherence, relevance, specificity, conciseness, professional vocabulary, organization, and persuasiveness, and the comments on vocal delivery were formative and independent of the acoustic classifications later obtained through the iMotions Voice Analysis module. Chatbot-generated scores were used exclusively as formative feedback and were not treated as validated psychometric measures or as outcome variables; the primary outcomes were the facial and vocal indicators obtained during the pre-training and post-training simulated interviews.

Calibration followed an iterative sequence of expert review of the questions and evaluation criteria; trial interactions to verify the coherence and professional relevance of the feedback; and adjustments to wording, tone, and scoring logic. The final prompt and question structure were fixed before the intervention so that all participants received a comparable training experience (Table 1).

### 3.5. Procedure and Intervention

The intervention consisted of a pre-training assessment, three chatbot-based training sessions, and a post-training assessment (Table 2).

First, participants completed a baseline simulated interview before receiving chatbot-based training. During this pre-training assessment, students answered a standard set of interview questions. Facial-expression recognition and vocal tone analysis technologies were used to record the initial facial and vocal emotional indicators.

Second, students participated in three chatbot-based training sessions. In each session, they answered five interview questions and received end-of-session numerical and qualitative feedback from the chatbot after completing the five-question sequence. The feedback addressed the clarity, coherence, vocabulary, conciseness, suitability of the responses, and, when voice input was used, observable aspects of vocal delivery. The second and third sessions allowed participants to apply the feedback received in previous interactions and progressively refine their answers.

Third, participants completed a post-training simulated interview. This final assessment followed the same general structure as the baseline interview. Facial-expression recognition and vocal analysis were again used to obtain the post-training facial and vocal emotional reaction time indicators.

Each pre-training and post-training simulated interview consisted of the same five standardized questions and concluded after the fifth answer. No fixed response time was imposed; interviews lasted approximately 5 min (range 3–7 min). Because the outcome variables were expressed as percentages of valid analyzable video time (facial) or of valid and intelligible speech time (vocal), differences in interview length were normalized within each modality.

After the post-training assessment, the course instructors, who also manage a recruitment company, retrospectively reviewed the interview recordings and provided formative observations. These observations were not delivered between the two measurement occasions and were not included as quantitative outcome measures; the chatbot should therefore be understood as a complementary training tool rather than a substitute for human expert judgment.

### 3.6. Laboratory Technologies and Data Collection

This study was conducted in an Applied Neuroscience Laboratory equipped with multimodal behavioral-analysis technologies; the outcomes reported here were obtained using iMotions 11.1.7 (iMotions A/S, Copenhagen, Denmark), with the Affectiva Affdex engine (Affectiva, Boston, MA, USA) for facial-expression analysis and the audEERING engine (audEERING GmbH, Gilching, Germany) for voice analysis (Table 3).

Facial-expression analysis was performed using the Affectiva Affdex engine integrated into iMotions 11.1.7 software. The system detects facial landmarks and facial-muscle movements associated with Action Units and generates confidence-based scores for facial-expression categories, including joy, anger, surprise, fear, sadness, disgust, and contempt, among others. Affectiva scores range from 0 to 100 and represent the model’s confidence that a particular visible expression is present. They should therefore be interpreted as indicators of observable facial configurations rather than direct measures of subjective emotional experience.

Although the laboratory is equipped with eye-tracking technology integrated into the iMotions platform, no calibrated eye-tracking data were collected or analyzed in the present study, and no claims regarding participants’ visual attention are made.

Vocal-expression analysis was performed using the iMotions Voice Analysis module with the audEERING engine, which analyzes acoustic and prosodic characteristics (pitch, loudness, speaking rate, and intonation) to classify speech segments into the categories happy, angry, sad, and neutral, reported here as happiness, anger, sadness, and neutrality. These categories are model-based estimates of vocal expression and not direct measures of psychological states such as anxiety, emotional regulation, or confidence.

Both Affectiva Affdex and the audEERING engine are proprietary AI-based systems. The researchers did not have access to the complete model architecture, training datasets, or internal weighting procedures underlying their classifications. Accordingly, the resulting outputs should be interpreted as software-dependent, algorithmically generated indicators of observable facial- and vocal-expression patterns rather than as direct or universally valid measures of underlying emotional states.

The Facial Emotional Reaction Time Proportion (%) was calculated as the percentage of valid analyzable video time during which the corresponding Affectiva Affdex category met the predefined detection criteria, and the Vocal Emotional Reaction Time Proportion (%) as the percentage of valid and intelligible speech time classified within the corresponding vocal category. Facial Emotion Analysis thresholding was enabled in iMotions, with a prespecified threshold of 25 applied identically to all participants, measurement occasions, and facial categories: a facial category was considered detected when its confidence score reached or exceeded 25, positive valence was defined as a valence score equal to or greater than 25, and negative valence as a score equal to or lower than −25; intermediate valence scores were treated as not meeting either criterion. The aggregate positive and negative emotional reaction time indicators were derived from positive- and negative-valence detections, respectively. The cutoff was an operational detection rule and not a clinically validated threshold.

The simulated interviews were recorded in high-definition video under stable lighting conditions, with microphones positioned close to the participants, and converted to MP4 format for processing in the iMotions platform.

### 3.7. Data Quality Control, Missing Data, and Detection Handling

Before processing in iMotions, all pre-training and post-training recordings underwent a structured audiovisual quality-control review conducted by the laboratory team. Each file was checked to verify that the complete five-question interview had been recorded, that the participant’s face remained sufficiently visible for facial landmark detection, that lighting was adequate and stable, that speech was clearly intelligible, and that the file had been correctly converted to MP4 format without corruption or playback errors. Compatibility with the iMotions platform was also verified before the recordings were included in the processing stage.

All recordings included in the final sample passed these quality-control checks. Consequently, no pre-training or post-training recording was excluded because of inadequate facial visibility, insufficient lighting, unintelligible audio, file corruption, or software incompatibility. All 54 participants provided complete paired recordings and valid values for the facial and vocal indicators required for the pre–post comparisons. Therefore, the final analytical sample consisted of 54 complete participant pairs.

No participant-level values were missing in the final analytical dataset, and no statistical imputation, interpolation, or replacement of missing values was required. Likewise, no participant was removed through listwise or pairwise deletion. All inferential analyses were performed using the same 54 participants assessed before and after the chatbot-based training.

At the signal-processing level, values that did not meet the predefined detection criteria were not treated as missing or ambiguous data. For the facial modality, an emotion or facial expression was classified as detected only when the corresponding Affectiva Affdex confidence score reached or exceeded the threshold of 25. Scores below 25 were interpreted as the absence of a threshold-level detection for that category, rather than as missing values. Similarly, valence scores between −25 and 25 were classified as not meeting the predefined criteria for positive or negative valence.

Vocal analyses were based on valid and intelligible speech segments processed by the audEERING Voice Analysis module. Because the preliminary review confirmed adequate audio clarity across the recordings included in this study, no interview was excluded because of unresolved vocal-signal quality problems. Facial percentages were calculated relative to valid analyzable video time, whereas vocal percentages were calculated relative to valid and intelligible speech time.

### 3.8. Variables and Measures

The main outcome variables were the Facial Emotional Reaction Time Proportion (%) and the Vocal Emotional Reaction Time Proportion (%). The participant was the unit of analysis, and each participant provided one pre-training and one post-training value for every facial and vocal indicator.

The Facial Emotional Reaction Time Proportion (%) represented the percentage of valid analyzable video time during which the corresponding facial category was detected by the Affectiva Affdex system. Facial outcomes were analyzed at two levels. Aggregate facial indicators included positive emotional reaction time, negative emotional reaction time, confusion, sentimentality, and neutral emotional reaction time. Specific facial-expression indicators included joy, surprise, anger, sadness, disgust, fear, and contempt.

The Vocal Emotional Reaction Time Proportion (%) represented the percentage of valid and intelligible speech time during which the audEERING Voice Analysis module classified speech within the corresponding vocal category. Vocal indicators included happiness, sadness, anger, and neutrality.

Higher values indicated that a greater proportion of the relevant analyzable time was classified within the corresponding category. Higher or lower percentages should not automatically be interpreted as better or worse interview performance, because the appropriateness of a given expression depends on the content and communicative context of the response; the indicators are model-based classifications of observable expression patterns rather than direct measurements of internal emotional states (Table 4).

### 3.9. Data Analysis

Statistical analyses were conducted using Stata/SE version 17.0 (StataCorp LLC, College Station, TX, USA). Because the same 54 participants were assessed at both measurement points and no data were missing, all analyses were conducted as paired comparisons based on 54 complete participant pairs.

Descriptive statistics were calculated for each outcome. Pre-training and post-training means were reported, and paired mean change was consistently defined as the post-training value minus the pre-training value. Accordingly, positive changes indicate increases after training, and negative changes indicate decreases. Mean changes are expressed in percentage points.

Paired-samples *t*-tests were used to assess mean pre–post differences. For each paired comparison, the mean change and its two-sided 95% confidence interval were reported. The standardized magnitude of the mean change was quantified using Cohen’s *d_z_*, calculated as the mean of the paired post-minus-pre differences divided by the standard deviation of those paired differences. Two-sided 95% confidence intervals for Cohen’s *d_z_* were calculated using the normal-approximation standard error, $SE_{d_{z}}=\sqrt{1/N+d_{z}^{2}/\left(2N\right)}$, with the lower and upper confidence limits calculated as $d_{z}\pm 1.96SE_{d_{z}}$.

Wilcoxon signed-rank tests were conducted as complementary rank-based paired analyses because several outcomes were bounded percentages, asymmetric, or concentrated near zero. For each Wilcoxon comparison, the effect-size statistic, *r*, was calculated as $Z/\sqrt{N}$, where *N* = 54 paired observations.

All statistical tests were two-sided. Conventional statistical significance was defined as *p* < 0.05. Values satisfying 0.05 ≤ *p* ≤ 0.10 were identified as marginal statistical evidence. Significance symbols were defined as † 0.05 ≤ *p* ≤ 0.10, * *p* < 0.05, ** *p* < 0.01, and *** *p* < 0.001.

Results were interpreted by jointly considering statistical significance; the direction and absolute magnitude of the paired mean change; its confidence interval; Cohen’s *d_z_*; the Wilcoxon effect-size statistic, *r*; and convergence between the paired-samples *t*-test and Wilcoxon signed-rank test. Statistical significance was not interpreted as equivalent to a large or practically important effect. When only the Wilcoxon signed-rank test was statistically significant, the finding was interpreted as evidence of a rank-based directional change rather than as evidence of a large average mean difference.

Given the absence of a control group, the analyses identify within-participant pre–post changes associated with chatbot-based training and do not establish that the intervention alone caused the observed differences.

## 4. Results

This section reports pre-training and post-training results for Facial and Vocal Emotional Reaction Time Proportions during simulated job interviews. The analyses examine within-participant changes associated with AI-powered chatbot-based training. The outcomes represent algorithmically classified facial- and vocal-expression patterns and should not be interpreted as direct measures of emotional regulation or psychological state.

Pre–post differences were evaluated using paired-samples *t*-tests and complementary Wilcoxon signed-rank tests. Table 5 and Table 6 report pre-training and post-training means; paired mean changes with two-sided 95% confidence intervals; paired-samples t statistics and *p*-values; Cohen’s *d_z_* with 95% confidence intervals; Wilcoxon Z statistics and *p*-values; and Wilcoxon effect-size estimates, *r*.

Results were interpreted by considering statistical significance together with the direction and magnitude of change, confidence-interval precision, effect-size estimates, and convergence between the mean-based and rank-based analyses.

All 54 participants contributed complete pre-training and post-training recordings, and all paired observations were retained in the final analyses. No participant or recording was excluded because of missing data or unresolved audiovisual quality problems.

Section 4.1 reports facial emotional reaction time, including aggregate indicators and specific facial-expression categories, and Section 4.2 reports vocal emotional reaction time (happiness, sadness, anger, and neutrality), in relation to the research question and the four hypotheses.

### 4.1. Facial Emotional Reaction Time Proportions in Simulated Job Interviews

Table 5 presents the paired pre–post comparisons for aggregate and specific facial emotional reaction time indicators. The outcomes represent algorithmically classified facial-expression patterns rather than direct measurements of internal emotional states, psychological readiness, or interview performance.

Positive facial emotional reaction time and facial joy showed the clearest facial changes, increasing by 11.23 and 7.72 percentage points, respectively. Both outcomes were statistically significant in the paired-samples *t*-test and the Wilcoxon signed-rank test, with Cohen’s *d*_z_ = 0.48, 95% CI [0.19, 0.76], for each outcome and Wilcoxon effect-size estimates of *r* = 0.42 and *r* = 0.40, respectively.

Several negative and neutral facial indicators decreased after training. However, their mean-based standardized effects were generally small or negligible, and some statistically significant findings were supported primarily by the Wilcoxon signed-rank test. These results are therefore interpreted as rank-based directional changes rather than as uniformly large average reductions.

Overall, the facial findings support H1 and provide partial support for H3 and H4.

#### 4.1.1. Positive Emotional Expressions

Positive facial emotional reaction time increased from 3.52% before training to 14.75% after training. The paired mean increase was 11.23 percentage points, 95% CI [4.79, 17.67], and was statistically significant, *t*(53) = 3.50, *p* < 0.001. The standardized paired effect was *d_z_* = 0.48, 95% CI [0.19, 0.76]. The complementary Wilcoxon signed-rank test was also statistically significant, *Z* = 3.05, *p* = 0.002, with an effect-size estimate of *r* = 0.42.

Facial joy showed a similar pattern, increasing from 2.38% to 10.10%. The paired mean increase was 7.72 percentage points, 95% CI [3.30, 12.14], *t*(53) = 3.50, *p* < 0.001, with *d_z_* = 0.48, 95% CI [0.19, 0.76]. The Wilcoxon signed-rank test also indicated a statistically significant increase, *Z* = 2.93, *p* = 0.003, *r* = 0.40. Thus, positive facial emotional reaction time and facial joy showed the clearest facial changes, with statistically significant results in both analyses and standardized effects approaching moderate magnitude.

Surprise remained practically unchanged, decreasing from 7.48% to 7.45%. The paired mean change was −0.03 percentage points, 95% CI [−0.12, 0.06], *t*(53) = −0.68, *p* = 0.500, *d_z_* = −0.09, 95% CI [−0.36, 0.18]. The Wilcoxon analysis was also not statistically significant, *Z* = −0.12, *p* = 0.904, *r* = −0.02.

Fear decreased from 6.61% to 6.11%, but the paired mean change was small and imprecisely estimated: −0.50 percentage points, 95% CI [−6.60, 5.60], *t*(53) = −0.16, *p* = 0.870, *d_z_* = −0.02, 95% CI [−0.29, 0.24]. The Wilcoxon test was also not statistically significant, *Z* = −1.37, *p* = 0.171, *r* = −0.19.

Anger increased slightly from 0.66% to 0.84%. The paired mean change was 0.18 percentage points, 95% CI [−0.59, 0.95], *t*(53) = 0.47, *p* = 0.640, *d_z_* = 0.06, 95% CI [−0.20, 0.33]. The Wilcoxon result was also not statistically significant, *Z* = 0.62, *p* = 0.535, *r* = 0.08.

#### 4.1.2. Negative Emotional Expressions

Negative facial emotional reaction time decreased from 1.82% before training to 0.70% after training. The paired mean change was −1.12 percentage points, 95% CI [−2.33, 0.09], *t*(53) = −1.85, *p* = 0.070, with *d_z_* = −0.25, 95% CI [−0.52, 0.02]. Thus, the paired-samples *t*-test provided marginal statistical evidence rather than conventional statistical significance. The Wilcoxon signed-rank test indicated a statistically significant rank-based reduction, *Z* = −2.58, *p* = 0.009, *r* = −0.35.

Facial sadness decreased from 0.48% to 0.30%. The paired mean change was −0.18 percentage points, 95% CI [−0.68, 0.32], *t*(53) = −0.73, *p* = 0.470, with *d_z_* = −0.10, 95% CI [−0.37, 0.17]. Although the mean-based comparison was not statistically significant, the Wilcoxon test indicated a statistically significant rank-based reduction, *Z* = −2.67, *p* = 0.008, *r* = −0.36.

Disgust decreased from 0.45% to 0.06%. The paired mean change was −0.39 percentage points, 95% CI [−0.86, 0.08], *t*(53) = −1.67, *p* = 0.100, with *d_z_* = −0.23, 95% CI [−0.50, 0.04]. The paired-samples *t*-test provided marginal statistical evidence, whereas the Wilcoxon test indicated a statistically significant rank-based reduction, *Z* = −2.19, *p* = 0.029, *r* = −0.30.

Contempt decreased from 3.34% to 0.62%. The paired mean change was −2.72 percentage points, 95% CI [−5.31, −0.13], and was statistically significant, *t*(53) = −2.11, *p* = 0.040. The standardized paired effect was small, *d_z_* = −0.29, 95% CI [−0.56, −0.01]. The Wilcoxon signed-rank test also indicated a statistically significant reduction, *Z* = −2.11, *p* = 0.035, *r* = −0.29.

Overall, contempt showed statistically significant reductions in both the mean-based and rank-based analyses, although the standardized effect was small. Negative facial emotional reaction time, facial sadness, and disgust were supported more clearly by the Wilcoxon analyses than by the paired mean comparisons. These findings therefore indicate rank-based directional reductions rather than uniformly large average effects.

#### 4.1.3. Neutral and Confusion Expressions

Neutral facial emotional reaction time decreased from 83.17% before training to 70.18% after training. The paired mean change was −12.99 percentage points, 95% CI [−25.98, 0.00], and was statistically significant, *t*(53) = −2.01, *p* = 0.049. The standardized paired effect was small, *d_z_* = −0.27, 95% CI [−0.54, 0.00]. The Wilcoxon signed-rank test also indicated a statistically significant reduction, *Z* = −2.08, *p* = 0.038, *r* = −0.28.

Confusion decreased from 1.07% to 0.70%. The paired mean change was −0.37 percentage points, 95% CI [−1.78, 1.04], *t*(53) = −0.53, *p* = 0.600, with *d_z_* = −0.07, 95% CI [−0.34, 0.20]. Although the mean-based comparison was not statistically significant, the Wilcoxon signed-rank test indicated a statistically significant rank-based reduction, *Z* = −2.42, *p* = 0.016, *r* = −0.33.

The reduction in neutral facial emotional reaction time was supported by both analyses, although its standardized effect was small. The reduction in confusion was supported only by the Wilcoxon analysis and should therefore be interpreted as a rank-based directional change rather than as a large average reduction. Lower neutral or confusion classifications should not automatically be interpreted as improved interview performance, because their meaning depends on the content and communicative context of the response.

#### 4.1.4. Sentimentality

Sentimentality increased from 1.09% before training to 2.18% after training. The paired mean change was 1.09 percentage points, 95% CI [0.00, 2.18], and was statistically significant, *t*(53) = 2.01, *p* = 0.049. The standardized paired effect was small, *d_z_* = 0.27, 95% CI [0.00, 0.54]. The Wilcoxon signed-rank test also indicated a statistically significant increase, *Z* = 2.01, *p* = 0.044, *r* = 0.27.

Thus, sentimentality showed a small but statistically significant increase in both the mean-based and rank-based analyses. This result should be interpreted as a change in an algorithmically classified facial-expression indicator and not as direct evidence of greater emotional openness, sincerity, interpersonal sensitivity, or improved interview performance.

### 4.2. Vocal Emotional Reaction Time Proportions in Simulated Job Interviews

Table 6 presents the paired pre–post comparisons for vocal emotional reaction time indicators. These outcomes represent algorithmically classified vocal-expression patterns and should not be interpreted as direct measurements of confidence, emotional stability, emotional regulation, or interview performance.

Vocal happiness showed the clearest vocal change, increasing by 7.92 percentage points, 95% CI [3.38, 12.46]. The change was statistically significant in both the paired-samples *t*-test and the Wilcoxon signed-rank test, with Cohen’s *d*_z_ = 0.48, 95% CI [0.19, 0.76], and a Wilcoxon effect-size estimate of *r* = 0.44.

Vocal sadness, anger, and neutrality decreased after training. However, their mean-based standardized effects were small, and the statistically significant findings were supported primarily by the Wilcoxon signed-rank tests. These results are therefore interpreted as rank-based directional reductions rather than as large average changes.

Overall, the vocal findings support H2 and provide partial support for H3 and H4.

#### 4.2.1. Vocal Happiness

Vocal happiness increased from 2.79% before training to 10.71% after training. The paired mean increase was 7.92 percentage points, 95% CI [3.38, 12.46], and was statistically significant, *t*(53) = 3.50, *p* < 0.001. The standardized paired effect was *d_z_* = 0.48, 95% CI [0.19, 0.76]. The Wilcoxon signed-rank test also indicated a statistically significant increase, *Z* = 3.21, *p* = 0.001, with an effect-size estimate of *r* = 0.44.

Vocal happiness therefore showed the clearest vocal change after training, with statistically significant results in both the mean-based and rank-based analyses and standardized effects approaching moderate magnitude. This finding represents an increase in an algorithmically classified vocal-expression category and should not be interpreted as direct evidence of greater confidence, enthusiasm, emotional regulation, or improved interview performance.

#### 4.2.2. Vocal Neutrality

Vocal neutrality decreased from 66.67% before training to 62.72% after training. The paired mean change was −3.95 percentage points, 95% CI [−8.68, 0.78], *t*(53) = −1.67, *p* = 0.100, with *d_z_* = −0.23, 95% CI [−0.50, 0.04]. The paired-samples *t*-test therefore provided marginal statistical evidence rather than conventional statistical significance. The Wilcoxon signed-rank test indicated a statistically significant rank-based reduction, *Z* = −1.97, *p* = 0.049, *r* = −0.27.

The reduction in vocal neutrality was supported primarily by the Wilcoxon analysis and should be interpreted as a small rank-based directional change rather than as a large average reduction. Lower vocal neutrality should not automatically be interpreted as improved interview performance, because neutrality may reflect composure, deliberation, professional restraint, or an individual communication style depending on the context.

#### 4.2.3. Vocal Sadness

Vocal sadness decreased from 13.84% before training to 12.71% after training. The paired mean change was −1.13 percentage points, 95% CI [−3.80, 1.54], *t*(53) = −0.85, *p* = 0.400, with *d_z_* = −0.12, 95% CI [−0.38, 0.15]. The paired-samples *t*-test was not statistically significant. However, the Wilcoxon signed-rank test indicated a statistically significant rank-based reduction, *Z* = −1.97, *p* = 0.049, *r* = −0.27.

The reduction in vocal sadness was therefore supported only by the Wilcoxon analysis and should be interpreted as a small rank-based directional change rather than as a statistically significant average reduction. This finding does not provide direct evidence of reduced insecurity, greater confidence, or improved emotional self-regulation.

#### 4.2.4. Vocal Anger

Vocal anger decreased from 1.64% before training to 1.41% after training. The paired mean change was −0.23 percentage points, 95% CI [−1.10, 0.64], *t*(53) = −0.53, *p* = 0.600, with *d_z_* = −0.07, 95% CI [−0.34, 0.20]. The paired-samples *t*-test was not statistically significant. However, the Wilcoxon signed-rank test indicated a statistically significant rank-based reduction, *Z* = −2.46, *p* = 0.014, *r* = −0.33.

The reduction in vocal anger was therefore supported only by the Wilcoxon analysis and should be interpreted as a rank-based directional change rather than as a statistically significant average reduction. This finding does not provide direct evidence of greater composure, improved emotional control, or better interview performance.

Overall, vocal happiness showed the clearest vocal change, with statistically significant results in both analyses and a standardized paired effect approaching moderate magnitude. Reductions in vocal sadness, anger, and neutrality showed small mean-based effects and were supported primarily by the Wilcoxon signed-rank tests.

## 5. Discussion

The findings of this study suggest that AI-powered chatbot-based training was associated with changes in the facial- and vocal-expression categories detected during simulated job interviews. Participants exhibited a greater proportion of detected facial joy and vocal happiness after training, together with reductions in several neutral and negatively classified categories. These findings describe changes in observable and algorithmically classified expression patterns; they do not, by themselves, demonstrate improved emotional regulation, communication competence, professional readiness, or interview success.

The interpretation of neutrality requires particular caution: a reduction in neutral classification may indicate greater expressive variation or engagement, but neutrality is not inherently deficient and may reflect composure, careful deliberation, professional restraint, or an individual communication style. Similarly, increases in joy or vocal happiness are not universally advantageous, because their appropriateness depends on the content and emotional demands of each response.

The observed changes are consistent with the iterative practice and feedback process implemented during the intervention. This cycle of practice, reflection, and adjustment, consistent with Kolb (2015) and with the conception of feedback as active cognitive and affective processing (Lipnevich & Panadero, 2021), allowed participants to identify areas for improvement and modify their responses across successive iterations, progressively reducing the gap between their initial responses and the expected standard (Hattie & Timperley, 2007).

Consequently, the observed pre–post changes are best interpreted as a shift in expressive behavior rather than as evidence that participants became more emotionally regulated or professionally competent. Determining whether these changes improve the quality of an interview would require external evaluations from trained recruiters, independent ratings of verbal and nonverbal performance, participant-reported psychological measures, and evidence from real selection outcomes.

Because no control group was included, no causal conclusion can be drawn. Practice effects, repeated exposure to the interview format, greater familiarity with the questions, and increased comfort with the procedure remain plausible alternative explanations.

Importantly, the practical magnitude of the findings should be considered alongside statistical significance. The largest absolute changes were observed for positive facial emotional reaction time (+11.23 percentage points), facial joy (+7.72 percentage points), and vocal happiness (+7.92 percentage points). Each of these outcomes showed a standardized paired effect of Cohen’s *d*_z_ = 0.48, 95% CI [0.19, 0.76], with Wilcoxon effect-size estimates of *r* = 0.42, *r* = 0.40, and *r* = 0.44, respectively. These results indicate non-negligible within-sample changes in the algorithmically classified expression indicators, although their practical significance for actual interview quality, recruiter evaluations, or employment outcomes remains uncertain because these outcomes were not measured.

In contrast, most reductions in negative and neutral indicators showed small or negligible mean-based standardized effects. Several nevertheless yielded statistically significant Wilcoxon results, including negative facial emotional reaction time (*r* = −0.35), confusion (*r* = −0.33), facial sadness (*r* = −0.36), disgust (*r* = −0.30), vocal sadness (*r* = −0.27), and vocal anger (*r* = −0.33). This divergence indicates that some changes were more consistent in rank and direction across participants than large in terms of average standardized change. These findings should therefore be interpreted more cautiously than the increases in positive facial emotional reaction time, facial joy, and vocal happiness.

### 5.1. Changes in Facial-Expression Patterns and Experiential Learning

In the pre-training assessment, participants showed a predominantly neutral facial profile together with low proportions of several negatively classified facial categories. These patterns should not be interpreted as direct evidence of anxiety, emotional instability, or limited professional readiness, because these constructs were not measured.

The chatbot-based training provided repeated interview practice, standardized questions, and end-of-session numerical and qualitative feedback. This sequence is consistent with experiential learning, as participants completed an interview cycle, reviewed feedback, and applied possible adjustments in subsequent practice sessions. Nevertheless, the one-group design does not establish that the chatbot alone produced the observed changes.

The clearest facial findings were the increases in positive facial emotional reaction time and facial joy. Both outcomes were statistically significant in the paired-samples *t*-test and Wilcoxon signed-rank test and showed standardized paired effects of *d_z_* = 0.48. Facial contempt also decreased significantly in both analyses, although its standardized effect was small. Reductions in negative facial emotional reaction time, facial sadness, disgust, and confusion were supported more clearly by the Wilcoxon analyses and should be interpreted as rank-based directional changes rather than as large average reductions.

These findings indicate changes in algorithmically classified facial-expression patterns following the training period. They do not demonstrate improved emotional regulation, greater confidence, enhanced communicative competence, or superior interview performance.

### 5.2. Facial- and Vocal-Expression Patterns Across Modalities

Facial and vocal analyses provided complementary but distinct information about the expression patterns classified during the simulated interviews. Because this study did not statistically evaluate cross-modal coherence, correspondence between facial and vocal indicators should not be assumed.

The clearest vocal finding was the increase in vocal happiness from 2.79% before training to 10.71% after training. This change was statistically significant in both analyses, with *d_z_* = 0.48, 95% CI [0.19, 0.76], and *r* = 0.44. Vocal sadness, anger, and neutrality decreased, but their mean-based standardized effects were small and the statistically significant findings were supported primarily by the Wilcoxon signed-rank tests.

Across the two modalities, the most consistent findings were the increases in positive facial emotional reaction time, facial joy, and vocal happiness, which indicate greater variation in algorithmically classified expression patterns following the training period rather than direct evidence of improved emotional control, confidence, communicative competence, or interview performance.

The chatbot provided repeated, structured interview practice and end-of-session feedback, but the one-group design does not establish that the intervention alone caused the observed changes. External recruiter ratings, independent assessments of verbal and nonverbal performance, and real recruitment outcomes would be required to determine whether these facial and vocal changes translate into improved interview evaluations.

### 5.3. Implications for Human Resources and Educational Practice

The findings have practical implications for higher education and human resources training. Chatbot-based interview simulations may provide students with repeated and standardized opportunities to practice interview responses in a structured environment. This approach may be particularly useful in employability, human talent management, career development, and professional communication courses.

The principal value of this approach lies in its capacity to complement traditional instruction by extending opportunities for practice and formative feedback. It should not replace mentoring, recruiter feedback, or professional evaluation. Human guidance remains necessary to assess the relevance, clarity, authenticity, and contextual appropriateness of participants’ responses.

The observed changes in facial- and vocal-expression indicators suggest that repeated chatbot-based practice may be associated with changes in how participants present themselves during simulated interviews. However, this study did not directly measure communication competence, confidence, employability, interview readiness, recruiter evaluations, or recruitment outcomes. Therefore, practical claims should remain limited to the expression patterns examined.

Educational institutions considering this type of training should ensure that the chatbot uses expert-validated questions, standardized instructions, transparent feedback criteria, and appropriate safeguards for privacy and data protection. Potential algorithmic bias should also be considered, particularly when automated facial or vocal classification systems are applied to participants with different cultural, linguistic, and individual communication styles.

For human resources practice, AI-assisted simulations may be used as complementary preparation tools rather than as screening or selection mechanisms. Future studies should determine whether the observed facial and vocal changes persist over time and whether they are associated with evaluations made by trained recruiters, independent observers, or real selection outcomes.

## 6. Conclusions

This study found that AI-assisted chatbot practice was associated with statistically significant pre–post changes in several facial- and vocal-expression indicators during simulated job interviews. The clearest findings were increases in positive facial emotional reaction time, facial joy, and vocal happiness. These outcomes showed standardized paired effects of *d_z_* = 0.48, together with moderate Wilcoxon effect-size estimates ranging from *r* = 0.40 to *r* = 0.44. Several negative and neutral indicators also decreased; however, the magnitude and statistical consistency of these changes differed across outcomes. The chatbot was configured using expert-validated interview questions, standardized instructions, and a consistent feedback structure. Its role was to provide repeated interview practice and formative feedback; the facial and vocal indicators analyzed in this study were obtained separately through the laboratory measurement systems and constituted the principal pre–post outcomes.

These results describe a change in algorithmically classified expression patterns during simulated interviews and should not be interpreted as direct evidence of improved emotional regulation, professional readiness, communication competence, or interview success. The contribution of this study is therefore limited to showing that structured practice with a calibrated AI-powered chatbot may be associated with changes in students’ facial- and vocal-expression patterns, highlighting the potential of personalized AI-based feedback to support interview practice while emphasizing the need for careful interpretation of automated classifications.

## 7. Limitations and Future Studies

This study has several limitations. First, the one-group pretest–posttest design without a control group does not allow the observed changes to be attributed to the chatbot-based training alone; repeated exposure to the interview format, greater familiarity with the questions, or increased comfort with the laboratory setting may also have contributed.

Second, the sample comprised 54 students from a single private Latin American university, and the interviews were simulated, which limits the generalizability of the findings and may not reproduce the pressure, unpredictability, and interpersonal complexity of real job interviews.

Third, the outcomes were limited to facial and vocal emotional reaction time generated by proprietary AI-based systems (Affectiva Affdex and audEERING), whose training data and internal procedures were not accessible to the researchers. These classifications are probabilistic and context-dependent; were not validated against participant self-reports, trained FACS coders, facial electromyography, or expert interviewers; and do not capture the full complexity of interview performance. Eye-tracking data were not included, because the recordings did not contain participant-specific, calibrated, and synchronized gaze measurements.

Fourth, several outcomes showed different inferential patterns in the paired-samples *t*-test and the Wilcoxon signed-rank test, and multiple outcomes were examined without a formal multiplicity adjustment, which increases the family-wise risk of Type I error; individual tests should therefore be interpreted as exploratory and in conjunction with effect sizes, confidence intervals, and convergence across statistical approaches. Finally, the chatbot’s scoring system was not validated against external human evaluators.

Future research should (a) use randomized controlled designs that compare chatbot-based training with control conditions and with alternative preparation methods, such as instructor-led practice, peer-based mock interviews, or career coaching; (b) adopt longitudinal designs to determine whether the changes persist over time; (c) preregister a smaller set of primary outcomes together with a multiplicity-control strategy; (d) integrate calibrated eye-tracking; (e) validate the automated classifications and the chatbot scores against recruiter ratings in Spanish-speaking contexts; and (f) examine whether the changes observed in simulated interviews translate into real recruitment outcomes.

## Figures and Tables

**Table 1:** Chatbot calibration process.

Calibration Stage	Description	Purpose
Expert review of interview questions	Ten experts reviewed the five core interview questions	Ensure professional relevance, clarity, and realism
Validation of evaluation criteria	Experts reviewed the scoring criteria: clarity, coherence, conciseness, vocabulary, emotional tone, and adequacy	Align chatbot feedback with HR interview standards
Trial interactions	Pilot interactions were conducted to review chatbot responses and feedback quality	Identify inconsistencies in scoring or recommendations
Prompt adjustment	The base prompt was refined to standardize tone, structure, and evaluation logic	Ensure consistency across participants
Final calibration	The final prompt and question structure were fixed before the intervention	Preserve comparability in the training process

**Table 2:** Intervention sequence.

Phase	Activity	Measurement	Purpose
Pre-training assessment	Baseline simulated interview	Facial-expression recognition and vocal analysis	Establish baseline facial and vocal emotional reaction time indicators
Training session 1	Chatbot-based interview practice with feedback	Chatbot score and qualitative feedback	Introduce structured feedback and identify areas for improvement
Training session 2	Repeated chatbot-based practice	Chatbot score and qualitative feedback	Apply previous recommendations and refine answers
Training session 3	Repeated chatbot-based practice	Chatbot score and qualitative feedback	Reinforce learning and consolidate response strategies
Post-training assessment	Final simulated interview	Facial-expression recognition and vocal analysis	Compare post-training facial and vocal emotional reaction time indicators with baseline

**Table 3:** Laboratory technologies and analytical role.

Technology	Data Collected	Role in This Study	Main Use in the Analysis
iMotions-supported facial-expression recognition	Facial Emotional Reaction Time Proportion (%)	Detection of algorithmically classified facial-expression patterns during simulated interviews	Primary outcome measure for facial indicators
iMotions Voice Analysis with the audEERING engine	Vocal Emotional Reaction Time Proportion (%)	Classification of algorithmically identified vocal-expression patterns in participants’ valid and intelligible speech	Primary outcome measure for vocal indicators
AI-powered chatbot	Interview responses, individual scores, overall session score, and qualitative feedback	Training tool for structured interview practice	Intervention mechanism and formative feedback tool; not analyzed as a primary outcome

**Table 4:** Variables and measures.

Dimension	Indicator Type	Variables	Measurement Unit	Interpretation
Facial expression	Aggregate indicators	Positive emotional reaction time, negative emotional reaction time, confusion, sentimentality, and neutral emotional reaction time	Percentage of valid analyzable video time	Proportion of valid video time during which each aggregate facial indicator was detected
Facial expression	Specific facial-expression indicators	Joy, surprise, anger, sadness, disgust, fear, and contempt	Percentage of valid analyzable video time	Proportion of valid video time during which each specific facial-expression category was detected
Vocal expression	Vocal emotional indicators	Happiness, sadness, anger, and neutrality	Percentage of valid and intelligible speech time	Proportion of valid and intelligible speech time classified within each vocal category
Chatbot-based interaction	Formative training indicators	Individual response scores and overall session score	0–10 scale	Used for formative feedback during training; not analyzed as a primary outcome

**Table 5:** Paired pre–post comparisons of Facial Emotional Reaction Time Proportions following AI-powered chatbot-based training.

Outcome	Pre-Training Mean (%)	Post-Training Mean (%)	Mean Change [95% CI] (pp)	*t*(53)	*p*	Cohen’s *d_z_* [95% CI]	Wilcoxon *Z*	*p*	Wilcoxon *r*
**Aggregate facial emotional reaction indicators**									
Positive emotional reaction time	3.52	14.75	+11.23 [4.79, 17.67]	3.50 ***	<0.001	0.48 [0.19, 0.76]	3.05 **	0.002	0.42
Negative emotional reaction time	1.82	0.70	−1.12 [−2.33, 0.09]	−1.85 ^†^	0.070	−0.25 [−0.52, 0.02]	−2.58 **	0.009	−0.35
Confusion	1.07	0.70	−0.37 [−1.78, 1.04]	−0.53	0.600	−0.07 [−0.34, 0.20]	−2.42 *	0.016	−0.33
Sentimentality	1.09	2.18	+1.09 [0.00, 2.18]	2.01 *	0.049	0.27 [0.00, 0.54]	2.01 *	0.044	0.27
Neutral emotional reaction time	83.17	70.18	−12.99 [−25.98, 0.00]	−2.01 *	0.049	−0.27 [−0.54, 0.00]	−2.08 *	0.038	−0.28
**Specific facial emotional reaction indicators**									
Joy	2.38	10.10	+7.72 [3.30, 12.14]	3.50 ***	<0.001	0.48 [0.19, 0.76]	2.93 **	0.003	0.40
Surprise	7.48	7.45	−0.03 [−0.12, 0.06]	−0.68	0.500	−0.09 [−0.36, 0.18]	−0.12	0.904	−0.02
Anger	0.66	0.84	+0.18 [−0.59, 0.95]	0.47	0.640	0.06 [−0.20, 0.33]	0.62	0.535	0.08
Sadness	0.48	0.30	−0.18 [−0.68, 0.32]	−0.73	0.470	−0.10 [−0.37, 0.17]	−2.67 **	0.008	−0.36
Disgust	0.45	0.06	−0.39 [−0.86, 0.08]	−1.67 ^†^	0.100	−0.23 [−0.50, 0.04]	−2.19 *	0.029	−0.30
Fear	6.61	6.11	−0.50 [−6.60, 5.60]	−0.16	0.870	−0.02 [−0.29, 0.24]	−1.37	0.171	−0.19
Contempt	3.34	0.62	−2.72 [−5.31, −0.13]	−2.11 *	0.040	−0.29 [−0.56, −0.01]	−2.11 *	0.035	−0.29

*Note.* Pre-training and post-training means represent the percentage of valid analyzable video time during which the corresponding facial category was detected. Mean change was calculated as the post-training mean minus the pre-training mean and is expressed in percentage points. Positive values indicate increases after training, whereas negative values indicate decreases. Two-sided 95% confidence intervals are reported for the paired mean change and Cohen’s *d_z_*. Cohen’s *d_z_* was calculated as the mean paired post-minus-pre difference divided by the standard deviation of the paired differences. The Wilcoxon effect-size statistic was calculated as $r=Z/\sqrt{N}$, where *N* = 54 paired observations. The signs of the mean change, *t*, *d_z_*, *Z*, and *r*, follow the post-minus-pre direction. Significance symbols placed beside *t* or *Z* refer to the corresponding displayed two-sided *p*-value: ^†^ 0.05 ≤ *p* ≤ 0.10, * *p* < 0.05, ** *p* < 0.01, and *** *p* < 0.001. The dagger denotes marginal statistical evidence and should not be interpreted as statistical significance at the conventional 0.05 level. No symbol indicates *p* > 0.10. CI = confidence interval; pp = percentage points.

**Table 6:** Paired pre–post comparisons of Vocal Emotional Reaction Time Proportions following AI-powered chatbot-based training.

Outcome	Pre-Training Mean (%)	Post-Training Mean (%)	Mean Change [95% CI] (pp)	*t*(53)	*p*	Cohen’s *d_z_* [95% CI]	Wilcoxon *Z*	*p*	Wilcoxon *r*
Happiness	2.79	10.71	+7.92 [3.38, 12.46]	3.50 ***	<0.001	0.48 [0.19, 0.76]	3.21 **	0.001	0.44
Sadness	13.84	12.71	−1.13 [−3.80, 1.54]	−0.85	0.400	−0.12 [−0.38, 0.15]	−1.97 *	0.049	−0.27
Anger	1.64	1.41	−0.23 [−1.10, 0.64]	−0.53	0.600	−0.07 [−0.34, 0.20]	−2.46 *	0.014	−0.33
Neutrality	66.67	62.72	−3.95 [−8.68, 0.78]	−1.67 ^†^	0.100	−0.23 [−0.50, 0.04]	−1.97 *	0.049	−0.27

*Note.* Pre-training and post-training means represent the percentage of valid and intelligible speech time during which the corresponding vocal category was classified. Mean change was calculated as the post-training mean minus the pre-training mean and is expressed in percentage points. Positive values indicate increases after training, whereas negative values indicate decreases. Two-sided 95% confidence intervals are reported for the paired mean change and Cohen’s *d_z_*. Cohen’s *d_z_* was calculated as the mean paired post-minus-pre difference divided by the standard deviation of the paired differences. The Wilcoxon effect-size statistic was calculated as $r=Z/\sqrt{N}$, where *N* = 54 paired observations. The signs of the mean change, *t*, *d_z_*, *Z*, and *r*, follow the post-minus-pre direction. Significance symbols placed beside *t* or *Z* refer to the corresponding displayed two-sided *p*-value: ^†^ 0.05 ≤ *p* ≤ 0.10, * *p* < 0.05, ** *p* < 0.01, and *** *p* < 0.001. The dagger denotes marginal statistical evidence and should not be interpreted as statistical significance at the conventional 0.05 level. No symbol indicates *p* > 0.10. CI = confidence interval; pp = percentage points.

## Data Availability

The datasets generated and analyzed during the current study are not publicly available due to privacy restrictions but are available from the corresponding author on reasonable request. The data that can be provided will be provided in a de-identified manner.
